# *Fusarium* Cyclodepsipeptide Mycotoxins: Chemistry, Biosynthesis, and Occurrence

**DOI:** 10.3390/toxins12120765

**Published:** 2020-12-03

**Authors:** Monika Urbaniak, Agnieszka Waśkiewicz, Łukasz Stępień

**Affiliations:** 1 Plant-Pathogen Interaction Team, Department of Pathogen Genetics and Plant Resistance, Institute of Plant Genetics of the Polish Academy of Sciences, Strzeszyńska 34, 60-479 Poznań, Poland; 2 Department of Chemistry, Poznan University of Life Sciences, Wojska Polskiego 75, 60-625 Poznań, Poland; agat@up.poznan.pl

**Keywords:** phytopathogens, *Fusarium*, mycotoxin contamination, secondary metabolism, beauvericin, enniatin

## Abstract

Most of the fungi from the *Fusarium* genus are pathogenic to cereals, vegetables, and fruits and the products of their secondary metabolism mycotoxins may accumulate in foods and feeds. Non-ribosomal cyclodepsipeptides are one of the main mycotoxin groups and include beauvericins (BEAs), enniatins (ENNs), and beauvenniatins (BEAEs). When ingested, even small amounts of these metabolites significantly affect human and animal health. On the other hand, in view of their antimicrobial activities and cytotoxicity, they may be used as components in drug discovery and processing and are considered as suitable candidates for anti-cancer drugs. Therefore, it is crucial to expand the existing knowledge about cyclodepsipeptides and to search for new analogues of these compounds. The present manuscript aimed to highlight the extensive variability of cyclodepsipeptides by describing chemistry, biosynthesis, and occurrence of BEAs, ENNs, and BEAEs in foods and feeds. Moreover, the co-occurrence of *Fusarium* species was compared to the amounts of toxins in crops, vegetables, and fruits from different regions of the world.

## 1. Introduction

Fungi belonging to the *Fusarium* genus produce a wide range of secondary metabolites, including the non-ribosomal depsipeptide mycotoxins, such as beauvericins (BEAs), beauvenniatins (BEAEs), enniatins (ENNs), and their analogues [1,2,3,4]. BEAs, BEAEs, and ENNs were included in the cyclodepsipeptide group of compounds, often found in high concentrations in grains, crops, vegetables, fruits, and even eggs, as a result of fungal infection [5,6,7,8,9]. They are involved in plant-pathogen interaction and may lead to many plants′ diseases, which can be very dangerous for animals′ health, including humans [10,11,12,13,14]. For example, ENNs produced by *Fusarium* species may act synergistically as a phytotoxin complex, which causes wilt and necrosis of plant tissue [15]. Moreover, ENN B affects mouse embryo development by inducing the dosage-related apoptosis or necrosis in mouse blastocytes [16]. On the other hand, BEA demonstrated neurotoxic properties in mice. In higher concentrations (7.5 and 10 µM), it affected the skeletal muscle fibers [17].

Additionally, BEA has a harmful influence on the reproductive system. The progesterone synthesis in cumulus cells was decreased when exposed to BEA [18]. Moreover, BEA inhibited estradiol and progesterone synthesis in bovine granulosa cells [19]. Also, ENN B reduced progesterone, testosterone, and cortisol secretion in human adrenocortical carcinoma cells and modulated the expression of genes involved in steroidogenesis [20]. The cytotoxicity of cyclodepsipeptides (BEAs, BEAEs, ENNs) is related to their ionophoric properties [21,22,23]. Even at low concentrations, they possess the capacity of perforation of the cell membrane, which is associated with the induction of apoptotic cell death and disruption of extracellular regulated protein kinase (ERK) activity [24,25,26,27]. However, this ability does not exclude the capability of promoting the transport of cations such as K^+^, Na^+^, Mg^2+^, and Ca^2+^ through the membranes, which leads to the disturbance of cellular ionic homeostasis [28]. This cytotoxic effect on various human cancer cell lines also suggests the potential use of cyclodepsipeptides as anti-cancer drugs [22,29,30,31,32]. All cyclodepsipeptides (BEAs, BEAEs, ENNs) have been shown as compounds exhibiting numerous biological activities, such as antimicrobial, insecticidal, and antibiotic activity, towards *Mycobacterium tuberculosis* and *Plasmodium falciparum* (human malaria parasite) because of their potential to inhibit the cholesterol acyltransferase of microbial origin [30,33]. Furthermore, BEA can be used as a co-drug for fungal infections in humans because the combination of BEA and ketoconazole (an anti-fungal drug) enhances its antifungal activities [29,33,34,35]. BEA has been reported as a growth inhibitor of human-pathogenic bacteria, such as *Escherichia coli*, *Enterococcus faecium*, *Salmonella enterica*, *Shigella dysenteriae*, *Listeria monocytogenes*, *Yersinia enterocolitica*, *Clostridium perfringens*, and *Pseudomonas aeruginosa*. The chemical properties of cyclodepsipeptides may allow for the emergence of new pharmaceutical products with anti-inflammatory and antibiotic properties [33,36,37]. The studies have shown the divergent impact of cyclodepsipeptides on human health; still, further studies are needed to indicate the potential effects of BEAs, BEAEs, and ENNs on human health. Moreover, it is imperative to study new compounds of the cyclodepsipeptide group, along with their analogues, to better understand the relationships between their structure, diversity, and toxicity.

The aim of the review article was to highlight the diversity among *Fusarium* species with regard to biosynthesis of BEAs, BEAEs, and ENNs and the characteristics of the multi-domain non-ribosomal peptide synthase (NRPS), which catalyses the synthesis of cyclodepsipeptides mycotoxins.

## 2. Chemistry

**Figure 1:**
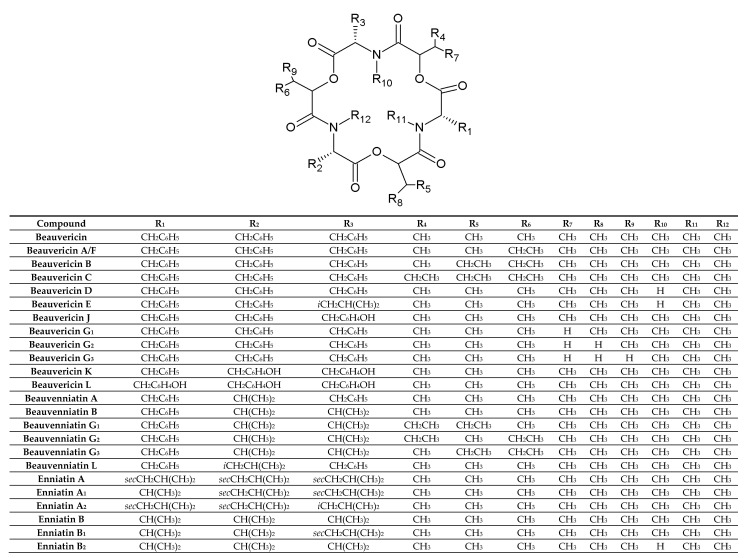

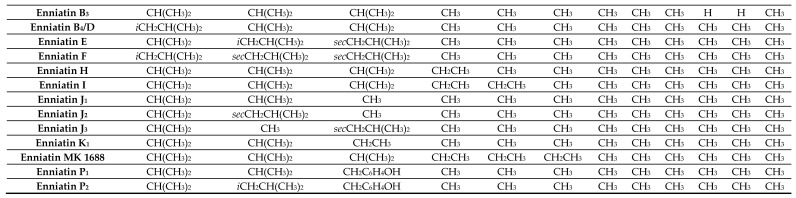
Chemical structures of beauvericin, enniatin, allobeauvericin, and beauvenniatin analogues produced by *Fusarium* species.

**Figure 2:**
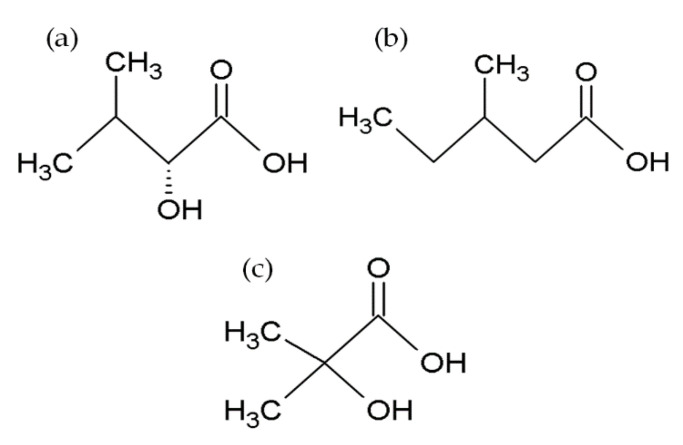
Chemical structures of D-2-hydroxyisovaleric acid (D-Hiv) (**a**), D-2-hydroxy-3-methylpentanoic acid (D-Hmp) (**b**), and D-2-hydroxybutyric acid (D-Hbu) (**c**) groups.

BEAs, ENNs, BEAEs, and allobeauvericins (ALLOBEAs) represent a family of regular cyclodepsipeptides, consisting of three *N*-methyl amino acids and three hydroxy acid groups [4,38,39,40,41]. Characterization of all cyclodepsipeptides produced by *Fusarium* fungi, their elemental composition, molecular weights (used for their identification), and chemical structures are presented in Table 1 and Figure 1. Most of the BEAs contain three groups of *N*-methyl-phenylalanine, except for BEAs J, K, and L, which contain one, two, or three groups of *N*-methyl-tyrosine, respectively [2,26]. However, BEA D and E have demethylated amino acids-phenylalanine and leucine in their structures [42]. Moreover, BEAs differ in hydroxy acids possession. BEA and BEA D, E, J, K, and L possess D-2-hydroxyisovaleric acid (D-Hiv) (Figure 2a) and BEA A/F, B, and C possess D-2-hydroxy-3-methylpentanoic acid (D-Hmp) (Figure 2b), whereas BEA G_1_ and G_2_ possess D-2-hydroxybutyric acid (D-Hbu) (Figure 2c) [2,3,31,33,42]. ALLOBEAs A, B, and C are diastereomeric to BEAs A, B, and C, respectively. These compounds differ in the D-Hmp groups’ configuration [33]. Some of the BEAs, such as BEA B, C, J, K, L, G_1_, G_2_, and all ALLOBEAs, were known from previous publications as precursor-directed compounds, detected inside in vitro cultures of fungi belonging to *Beauveria*, *Acremonium*, and *Paecilomyces* genera [26,31,33]. It was proven that phytopathogenic fungi from the *Fusarium* genus naturally produce all BEAs and ALLOBEAs [2,3,42]. The structures of BEAs have been described in many articles, where they were determined by a variety of chemical methods, including liquid chromatography–mass spectrometry (LC-MS) and nuclear magnetic resonance (NMR).

ENNs are typically composed of *N*-methyl-leucine, *N*-methyl-isoleucine and/or *N*-methyl-valine [1,10,41]. However, two of the ENNs: ENN P_1_ and P_2_ also possess *N*-methyl-tyrosine in their structures [21]. ENN J_1_, J_2_, and J_3_ are another group of ENNs that differ from the common ENNs. These cyclodepsipeptides consist of one *N*-methyl-isoleucine, one *N*-methyl-valine, and *N*-methyl-alanine [43]. Most ENNs contain three groups of D-2-hydroxyisovaleric acid (D-Hiv) and only three ENNs: ENN H, I, and MK 1688, containing one, two, or three groups of D-2-hydroxy-3-methylpentanoic acid (D-Hmp), respectively [44]. Some of the reported ENNs are isomers, with the same amino acid composition but in different positions, e.g., ENN J_1_, J_2_, J_3_ or ENN A and F [39,43,45]. On the other hand, even though the ENNs are not isomers, they share the same molecular weight. Therefore, the MS/MS technique with acid hydrolysis or NMR is sometimes necessary during the detection of cyclodepsipeptides for their correct identification.

BEAEs possess hybrid structures between the aliphatic (enniatin-type) and aromatic (beauvericin-type) cyclodepsipeptides [2,3,26,30]. Moieties of *N*-methyl-phenylalanine, *N*-methyl-leucine, and/or *N*-methyl-valine are the parts of BEAEs’ structures. BEAE A contains one *N*-methyl-valine, whereas BEAE B, G_1_, G_2_, and G_3_ have two. BEAE L has one *N*-methyl-leucine in its structure. Apart from the D-2-hydroxyisovaleric acid (D-Hiv) group, three of the BEAE isomers, namely BEAE G_1_, G_2_, and G_3_, contain two D-2-hydroxy-3-methylpentanoic acid (D-Hmp) groups in different combinations. At first, all BEAEs were described as cyclodepsipeptides from *Acremonium* sp., however further research revealed that *Fusarium* species are also able to produce these compounds [2,3,26,30].

## 3. Biosynthesis

Cyclodepsipeptides are biosynthesized by a multi-domain non-ribosomal peptide synthase (NRPS) that is composed of enzymatic modules used to elongate the proteinogenic and non-proteinogenic amino acids, as well as carboxyl and hydroxy acids [48,49]. The modules respond to the order and number of the precursors incorporated into the chain. Separate NRPS modules are required to assemble the product and a minimal module consists of the three core domains: adenylation (A) domain, thiolation or peptidyl-carrier protein (T or PCP) domain, and condensation (C) domain. Moreover, each module and each active site domain is used only once for the recognition and activation of the precursors through adenylation with ATP (A: adenylation domain), covalent thioester tethering (T: thiolation or PCP: peptidyl carrier protein domain), which tethers the activated precursor to a 4′-phosphopantetheine (PP) cofactor through a thioester bond and transport substrates to the active sites of the domains, and condensation (C domain) of the precursors via catalyzing the peptide bond (C-N) formation between the elongated chain and the activated amino acid. The main domains may be supported by additional domains of the NRPS, such as the epimerization (E) domain, which catalyzes the transformation of an L-amino acid into a D-amino acid or the dual/epimerization (E/C) domains, which catalyze the epimerization and condensation. NRPSs contain an additional reductase (R) domain, which is responsible for reducing the final peptide, the methylation (MT) domain, which catalyzes *N*-methylation of the amino acid substrate, the cyclization (Cy) domain that catalyzes the formation of oxazoline or thiazoline rings by internal cyclization of cysteine, serine, or threonine residues, and the oxidation (Ox) domain, which catalyzes the formation of an aromatic thiazol through oxidation of a thiazoline ring. The last domains (TE–thioesterase domains), mostly located at the final NRPS module, are responsible for releasing the full-length NRPS product from the enzyme through cyclization or hydrolysis [48,49,50,51,52].

**Figure 3:**
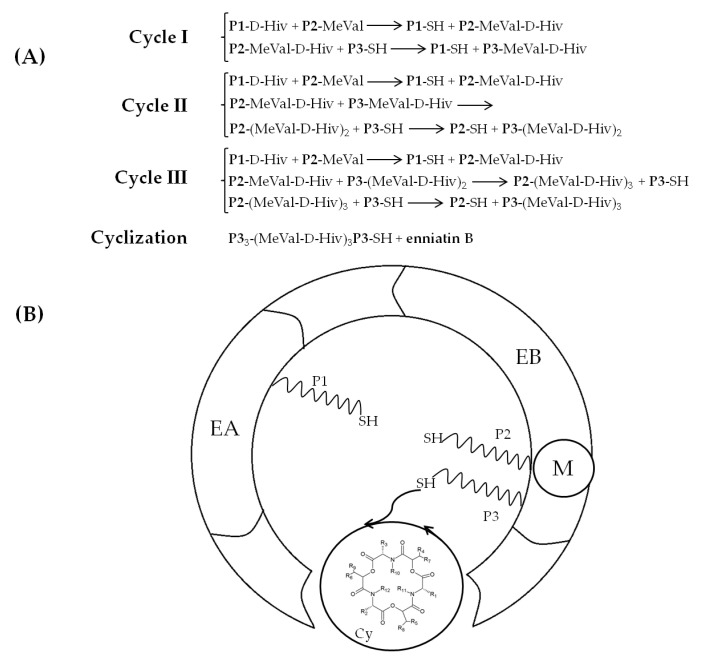
Mechanism of enniatin B formation according to Hornbogen et al. [4]. (**A**) Scheme of partial reactions leading to the formation of ENN B, P1, P2, P3 = 4′-phosphopantetheine. (**B**) Model of arrangement of catalytic sites in enniatin synthase; Cy: cyclization cavity; EA: D-Hiv-activation module; EB: L-valine-activation module; M: *N*-methyltransferase domain.

Enniatin biosynthesis is catalyzed by the 347 kDa multienzyme enniatin synthase (ESYN1) purified for the first time from *Fusarium oxysporum* and further characterized by Zocher and coworkers [53]. Extensive molecular research revealed the basis of cyclic oligopeptide biosynthesis and allowed us to identify *esyn1*, a gene encoding enniatin synthase, as the essential enzyme of the metabolic pathway [39,54,55,56,57]. The biochemical characterization revealed that the enzyme possesses two substrate activation modules EA and EB, composed of approximately 420 amino acid residues. The EA module activates and participates in binding the α-D-hydroxy acids, while the EB module activates the amino acids. These two modules consist of a conserved 4-phosphopantetheine binding site at the C-terminus, with a highly conserved serine residue. An additional 4-phosphopantetheine group and *N*-methyltransferase domain M are present in the EB module. Also, a putative condensation (C) domain exists between the EA and EB modules. The M domain is highly conserved among *N*-methyl peptide synthases of prokaryotic and eukaryotic origin, thus it represents only local sequence similarities to the structural elements of other AdoMet-dependent methyltransferases. A dipeptidol unit is formed due to the interaction between the EA and EB modules and later, it is transferred and condensed into a thiol group. Three such successive condensations of the enzyme-bound dipeptidols are followed by the ring′s closure into the enniatin (ENN) molecule [4,58,59,60,61] (Figure 3A,B).

The primary precursors of the ENNs are valine, leucine or isoleucine, D-2-hydroxyisovaleric acid, and S-adenosylmethionine and their synthesis is entirely dependent on the cyclization reaction of linear hexadepsipeptide. The amino acid specificity of ESYN1 contributes to the chemical diversity of ENNs and this is why different types of ENNs are produced by *Fusarium scirpi*, *F. lateritium*, and *F. sambucinum*. The Esyn domains activating L-valine in *F. scirpi* and preferably activating L-isoleucine in *F. sambucinum* are nearly identical, with an exception of the three regions showing significant differences in their structures. This difference in the activation can be accredited to the mutations that eventually occurred in the amino acid recognition sites of various enniatin synthases. In spite of the variability in amino acid units, certain ENNs can only be isolated from specific *Fusarium* strains, in which the enniatin synthase prefers some amino acids over others during biosynthesis [4,53,62,63,64,65].

**Figure 4:**
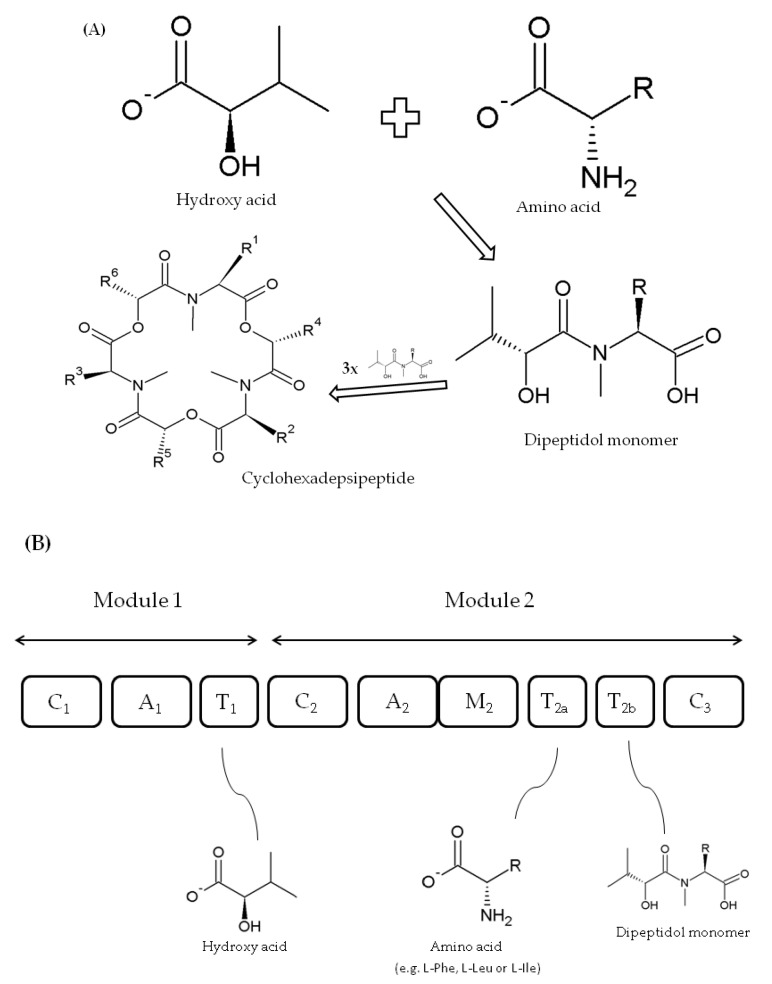
Biosynthesis of fungal cyclodepsipeptides (**A**) and model of beauvericins (BEAS) synthase structure with domain roles (domains not to scale) (**B**) according to Xu et al. [50,52].

BEAs are also formed as cyclic trimers assembled from three D-Hiv-*N*-methyl-L-amino acid dipeptidol monomers (Figure 4A) [50,51]. Similarly, they are also produced by a thiol template mechanism and synthesized by beauvericin synthase (BEAS) enzyme, which consists of a single polypeptide chain of about 351 kD [41,50]. For the first time, the 250 kDa BEAS enzyme was characterized by Peeters et al. [66] from the entomopathogenic fungus *Beauveria bassiana*, although Xu et al. [50], who conducted a more in-depth analysis, described a 33,475 bp beauvericin gene cluster including a 9570 bp *bbBeas* gene. Five years later, Zhang and coworkers [51] cloned and characterized 9413 bp beauvericin synthase gene (*fpBeas*) from *Fusarium proliferatum*.

The C_1_, A_1_, and T_1_ domains within the first module of FpBEAS and ESYN (EA module) synthases have the same role in cyclodepsipeptide formation [51]. Nevertheless, the two depsipeptide synthases differ in A_2_ domain substrate specificity within module 2 (ESYN EB module), i.e., apart from that of enniatin synthase, beauvericin synthase preferably accepts *N*-methyl-L-phenylalanine and some other aliphatic hydrophobic amino acids (e.g., leucine or isoleucine) [50]. Furthermore, their incorporation efficiency reduces with the length of side chains, where ortho-, meta-, and para-fluoro-substituted phenylalanine derivatives and *N*-methyl-L-leucine, *N*-methyl-L-norleucine, and *N*-methyl-L-isoleucine residues could replace *N*-methyl-L-phenylalanine. Domains C_2_, T_2a;b_, M_2_, and C_3_ within module 2 of BEAS and ESYN play the same role in both synthases (Figure 4B) [50,66].

**Figure 5:**
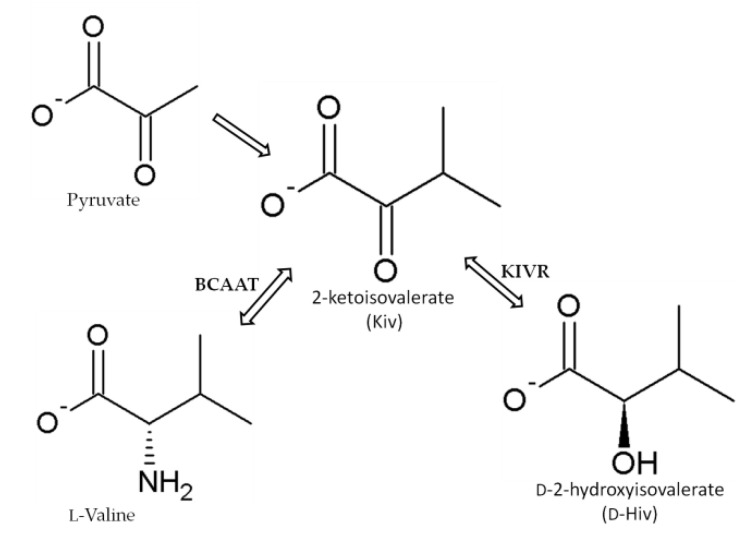
Synthesis of 2-ketoisovalerate (Kiv), a substrate used in the formation of D-2-hydroxyisovaleric acid (D-Hiv) moiety by 2-ketoisovalerate reductase (KIVR) according to Xu et al. [67]. BCAAT: branched-chain amino acid aminotransferase.

The depsipeptides, including BEAs, have a common 2-hydroxycarboxylic acid ingredient–D-2-hydroxyisovalerate (D-Hiv) that is formed from 2-ketoisovalerate (2-Kiv) by a highly specific chiral reduction reaction catalyzed by 2-ketoisovalerate reductase (KIVR) enzyme [50,52,67,68,69,70]. KIVR has a significant role in the biosynthesis of BEAs as was clearly understood when BEA production was inhibited in a KIVR knock-out *B. bassiana* mutant [67]. Kiv is formed from pyruvate during the biosynthesis of valine and it is the key intermediate in several metabolic pathways, including pantothenate biosynthesis in fungi, bacteria, and plants. It is also involved in producing phosphopantetheinyl prosthetic groups of acyl or peptidyl carrier proteins and co-enzyme A (Figure 5) [50,52,67,69,70].

Significant sequence homologies were identified for certain *Fusarium* enzymes, which shows a common genetic background for the synthesis of both depsipeptide compounds. Zhang et al. [51] revealed in their analysis that FpBEAS (GenBank acc. no. JF826561.1) has 64% identity to ESYN (GenBank acc. no. CAA79245) as it was proven that some *Fusarium* species, like *F. poae*, *F. proliferatum*, or *F. oxysporum* were found to produce ENNs and BEA simultaneously. This is justified by the fact that both toxins share a metabolic pathway [1,44,71,72]. Reports suggest that there is a high probability that the single PCR based *esyn1-* and/or *BEAS-* specific marker can detect potential BEAs and ENNs-producing fungi from contaminated soil and plant material [39,55,73].

## 4. *Fusarium* Species and Cyclodepsipeptide Mycotoxins in Food and Feed

Plant crops are critical mainly in terms of yield and diverse use for foods and feeds. They suffer from a range of fungal diseases and *Fusarium* species are among the most damaging pathogens, producing toxic secondary metabolites, such as cyclodepsipeptides. Cyclodepsipeptides biosynthesis has been observed for 44 *Fusarium* species (Table 2) and *F. acuminatum*, *F. concentricum*, *F. proliferatum*, *F. verticillioides*, *F. oxysporum*, and *F. tricinctum* produce a broad spectrum of ENN, BEA, and BEAE analogues. The remaining *Fusarium* species formed only individual mycotoxin groups, such as BEA, ENNs, or a mixture of these. However, in a few research papers, it was not specified which *Fusarium* species produced ENNs and the presence of mycotoxins was described as a “mix of ENNs” (Table 2).

*Fusarium* species can cause many plant diseases and one of them is *Fusarium* head blight (FHB), which is devastating for cereal species, particularly as it is a major problem regarding wheat production in many countries. Usually, one or more *Fusarium* species (*F. graminearum*, *F. culmorum*, *F. avenaceum*, *F. poae*, and *F. sporotrichioides*) are involved as causal agents [74]. The occurrence of many *Fusarium* species may increase the accumulation of mycotoxins in grains or plants and introduce them into the food chain [71,75,76]. Humidity and temperature determine the disease severity, but geographical conditions, plant genotype, and local pathogen populations also play essential roles [54,77].

Available literature data relate both to identifying *Fusarium* fungi isolated from various hosts and analyzing their mycotoxin biosynthesis capacity (Table 3). Efforts are also being made to assess contamination levels with these toxins of raw plant materials and food and feed products (Table 4). Mainly, the content of BEA and four ENNs (ENN A, ENN A_1_, ENN B, ENN B_1_) has been investigated [8,25]. BEA and ENNs are common contaminants and were detected in plant crops and grains throughout the world. The occurrence of BEA, ENN A, ENN A_1_, ENN B, and ENN B_1_ in naturally contaminated crops has been studied much more extensively than the occurrence of other cyclodepsipeptides [1,39]. Table 3 summarizes the most effective producers of depsipeptides among *Fusarium* fungi isolated from different crops and geographical areas. *F. avenaceum*, *F. equiseti*, *F. proliferatum*, and *F. sporotrichioides* were the most common species isolated from plants. The best producer of BEA was *F. proliferatum* (FPG61_CM), isolated from garlic in Spain, with the concentration reaching 671.80 μg/g [6]. The highest yielding producers of ENNs were *F. avenaceum* (KF1330), isolated from wheat in Poland, and *F. tricinctum* (3405), isolated from wheat in Finland [5,39]. Both strains produced in the highest amounts ENN B (895.46 μg/g, 690 μg/g) and ENN B_1_ (452.46 μg/g, 1200 μg/g) [5,39].

Table 4 presents the maximum amounts of BEA and ENNs in naturally contaminated plant crops described in the literature. The highest contamination level of BEA was found to be 1731.55 μg/g in Polish maize [95]. When compared to other cyclodepsipeptides, it was also the highest concentration of mycotoxin in crops. In Tunisian sorghum, maximum concentrations of ENN A (95.6 μg/g) and ENN B_1_ (120.1 μg/g) were detected [96]. The highest amount of ENN A_1_ was 813.01 μg/g and 814.42 μg/g in Spanish maize and rice, respectively [97]. ENN B was found with a maximum level of 180.6 μg/g in Tunisian wheat [96]. The data show very high variability of investigated cyclodepsipeptides and it can be concluded that each strain of *Fusarium* species possesses a unique ability to biosynthesize these compounds. In addition to crops, cyclodepsipeptides are also found in food and feed [98,99,100,101,102,103]. Cyclodepsipeptides were identified mainly in cereal food, with very high levels of ENN A_1_ and B_1_ in breakfast cereals from Morocco (668 and 795 μg/g, respectively) [99]. In feed samples, ENNs and BEA levels were very low and did not exceed 0.48 μg/g for BEA (poultry feed) and 2.19 μg/g for ENNs (poultry feed) [101].

## 5. Conclusions

Fungi from the *Fusarium* genus produce a unique set of cyclodepsipeptide analogues of different amounts. The described mycotoxins are involved in plant-pathogen interaction, thus they were detected in a range of foodstuffs or feeds originating from many countries. They may be very dangerous for human health because of their biological activities. On the other hand, cyclodepsipeptides possess antimicrobial, insecticidal, antifungal, and antibiotic activities, which may help develop new drugs. In addition, because of their cytotoxicity, cyclodepsipeptides may have applications in anti-cancer therapy. Moreover, new BEAs, ENNs, or BEAEs with different amino/hydroxy acid compositions are detected each year inside in vitro fungal cultures. It was proven that not only fungi from *Fusarium* genus naturally produce cyclodepsipeptides, but also other fungi belonging to *Beauveria*, *Acremonium*, and *Paecilomyces* genera. Therefore, it is essential to continually improve the knowledge regarding these compounds, their structure, diversity, and toxicity to screen products of fungal secondary metabolism and monitor the dispersion of phytopathogenic fungi, which are potent producers of threatening mycotoxins. Moreover, it would be beneficial to bettering the understanding of cyclodepsipeptide biosynthesis to investigate the diversity and evolution history of the BEAS/ESYN synthase gene cluster from various fungi.

## Figures and Tables

**Table 1:** Elemental composition and molecular weights of beauvericins, enniatins, and their analogues.

Compound	MW	MW + NH_4_^+^ (18)	MW + Na^+^ (23)	MW + K^+^ (39)	Elemental Composition	References
**Beauvericin**	783	801	806	822	C_45_H_57_N_3_O_9_	[2,26]
**Beauvericin A/F/Allobeauvericin A**	797	815	820	836	C_46_H_59_N_3_O_9_	[2,33,42]
**Beauvericin B/Allobeauvericin B**	811	829	834	850	C_47_H_61_N_3_O_9_	[3,33]
**Beauvericin C/Allobeauvericin C**	825	843	848	864	C_48_H_63_N_3_O_9_	[2,33]
**Beauvericin D**	769	787	792	808	C_44_H_55_N_3_O_9_	[2,42]
**Beauvericin E**	735	753	758	774	C_41_H_57_N_3_O_9_	[3,42]
**Beauvericin G_1_**	769	787	792	808	C_44_H_55_N_3_O_9_	[3,31]
**Beauvericin G_2_**	755	773	778	794	C_43_H_53_N_3_O_9_	[3,31]
**Beauvericin J**	799	817	822	838	C_45_H_57_N_3_O_10_	[2,26]
**Beauvericin K**	815	833	838	854	C_45_H_57_N_3_O_11_	[2]
**Beauvericin L**	831	849	854	870	C_45_H_57_N_3_O_12_	[2]
**Beauvenniatin A**	735	753	758	774	C_41_H_57_N_3_O_9_	[2,26]
**Beauvenniatin B**	687	705	710	726	C_37_H_57_N_3_O_9_	[3,26,30]
**Beauvenniatin G_1_/G_2_/G_3_**	715	733	738	754	C_39_H_61_N_3_O_9_	[3,30]
**Beauvenniatin L**	749	767	772	788	C_42_H_59_N_3_O_9_	[2]
**Enniatin A/F/MK 1688**	681	699	704	720	C_36_H_63_N_3_O_9_	[25,39,44,45]
**Enniatin A_1_/E/I**	667	685	690	706	C_35_H_61_N_3_O_9_	[25,39,44,45]
**Enniatin A_2_**	681	699	704	720	C_35_H_61_N_3_O_9_	[46]
**Enniatin B**	639	657	662	678	C_33_H_57_N_3_O_9_	[25,39]
**Enniatin B_1_/B_4_/D/H**	653	671	676	692	C_34_H_59_N_3_O_9_	[25,39,44,45,47]
**Enniatin B_2_/J_2_/J_3_/K_1_**	625	643	648	664	C_32_H_55_N_3_O_9_	[25,43]
**Enniatin B_3_/J_1_**	611	629	634	650	C_31_H_53_N_3_O_9_	[25,43,47]
**Enniatin P_1_**	641	659	664	680	C_33_H_57_N_3_O_10_	[21]
**Enniatin P_2_**	655	673	678	694	C_34_H_59_N_3_O_10_	[21]

**Table 2:** Cyclodepsipeptides mycotoxins produced by various *Fusarium* species.

*Fusarium* Species	Compound	References
***F. acuminatum***	BEA, ENN A, ENN A_1_, ENN B, ENN B_1_, ENN B_2_, ENN B_3_, ENN B_4_, ENN P_1_, ENN P_2_, BEA C, BEA D, BEA G_1_, ALLOBEA C	[2,3,5,21,39,47,78]
***F. acutatum***	BEA, mix of ENNs	[79]
***F. ananatum***	BEA, ENN A, ENN B, ENN B_1_	[39]
***F. anthophilum***	BEA, ENN A, ENN B, ENN B_1_	[39,78]
***F. arthrosporioides***	mix of ENNs	[15]
***F. avenaceum***	BEA, ENN A, ENN A_1_, ENN B, ENN B_1_, ENN B_2_, ENN B_3_, ENN B_4_	[25,39,78,80,81]
***F. beomiforme***	BEA	[78]
***F. bulbicola***	BEA	[79]
***F. circinatum***	BEA	[79,82]
***F. concentricum***	BEA, ENN A, ENN A_1_, ENN B, ENN B_1_, BEA A/F, BEA B, BEA C, BEA D, BEA E, BEA G_1_, BEA G_2_, BEA J, BEA K, BEA L, BEAE A, BEAE B, BEAE G_1_/G_2_/G_3_, BEAE L, ALLOBEA A, ALLOBEA B, ALLOBEA C	[2,3,39,79,82]
***F. compactum***	ENN A, ENN A_1_, ENN B, ENN B_1_, ENN B_2_	[47]
***F. culmorum***	mix of ENNs, ENN B	[83]
***F. denticulatum***	BEA	[79]
***F. dlamini***	BEA, ENN A, ENN A_1_, ENN B_1_	[39,78,79]
***F. equiseti***	BEA, ENN A, ENN A_1_, ENN B, ENN B_1_	[39,78]
***F. fujikuoi***	BEA	[79]
***F. globosum***	BEA	[84]
***F. guttiforme***	BEA	[79,82]
***F. graminearum***	ENN A, ENN A_1_, ENN B, ENN B_1_	[85]
***F. konzum***	BEA	[86]
***F. kyushuense***	ENN B, ENN B_1_	[87]
***F. lactis***	BEA, ENN A, ENN A_1_, ENN B, ENN B_1_	[39,79]
***F. langsethiae***	BEA, ENN A_1_, ENN B, ENN B_1_	[87]
***F. lateritium***	mix of ENNs	[15]
***F. longipes***	BEA	[78]
***F. merismoides***	mix of ENNs	[15]
***F. nygamai***	BEA, ENN A, ENN A_1_, ENN B	[39,78,79]
***F. oxysporum***	BEA, BEA A/F, BEA B, BEA C, BEA D, BEA E, BEA G_1_, BEA G_2_, BEA J, BEAE A, BEAE B, BEAE L, ALLOBEA A, ALLOBEA B, ALLOBEA C, ENN A_1_, ENN B, ENN B_1_, ENN H, ENN I, ENN MK1688	[2,3,39,44,78]
***F. poae***	BEA, ENN A, ENN A_1_, ENN B, ENN B_1_	[39,71,78,87]
***F. phyllophilum***	BEA	[79]
***F. proliferatum***	BEA, ENN A_1_, ENN B, ENN B_1_, BEA A/F, BEA B, BEA C, BEA D, BEA E, BEA G_1_, BEA G_2_, BEA J, BEA K, BEAE A, BEAE B, BEAE L, ALLOBEA A, ALLOBEA B, ALLOBEA C	[2,3,39,84]
***F. pseudoanthophilum***	BEA	[82]
***F. pseudocircinatum***	BEA	[79]
***F. redolens***	BEA	[37]
***F. sacchari***	BEA	[79]
***F. sambucinum***	BEA, mix of ENNs	[15,78]
***F. scirpi***	mix of ENNs	[15]
***F. semitectum***	BEA	[88]
***F. sporotrichioides***	BEA, ENN A, ENN B, ENN B_1_, ENN A_1_	[39,71,87]
***F. subglutinans***	BEA, ENN A, ENN B, ENN B_1_	[39,88,89,90]
***F. succisae***	BEA	[79]
***F. temperatum***	BEA, ENN A, ENN A_1_, ENN B, ENN B_1_	[39,90]
***F. torulosum***	ENN B	[91,92]
***F. tricinctum***	BEA, ENN A, ENN A_1_, ENN B, ENN B_1_, ENN B_4_, ENN J_1_	[5,36,39,93]
***F. verticillioides***	BEA, ENN B, ENN B_1_, BEA C, BEA D, BEA G_1_, BEA K, BEAE A, ALLOBEA C	[2,3,39,94]

“ENN”—enniatin; “BEA”—beauvericin; “ALLOBEA”—allobeauvericin; “BEAE”—beauvenniatin.

**Table 3:** The strains of *Fusarium* species from different origin and hosts, producing the highest amounts of cyclodepsipeptides [μg/g].

Species	ID Strain	Host	Origin	ENN A	ENN A_1_	ENN B	ENN B_1_	ENN B_2_	ENN B_3_	BEA	Analytical Method	Reference
***F. acuminatum***	KF 3713	Pea	Poland	19.62	26.92	90.89	31.49	NA	NA	5.31	HPLC	[39]
***F. ananatum***	KF 3557	Pineapple	Costa Rica	6.94	ND	8.81	27.60	NA	NA	27.68	HPLC	[39]
***F. avenaceum***	KF 3803	Asparagus	Poland	ND	≤0.01	0.03	ND	NA	NA	ND	HPLC	[39]
	11B14	Barley	Italy	10.9	193	45	172	55	1.58	NA	LC-MS/MS	[104]
	KF 3717	Pea	Poland	6.09	5.65	6.71	11.46	NA	NA	ND	HPLC	[39]
	Fa40	Wheat	Italy	165.8	109.2	35.5	60.2	NA	NA	ND	LC-DAD	[71]
	KF 1337	Wheat	Poland	34.55	71.90	895.46	452.46	NA	NA	ND	HPLC	[39]
	44	Wheat	Italy	7.24	34.3	6.6	17.8	0.67	≤0.01	≤0.01	LC-MS/MS	[105]
	Fa34	Wheat	Italy	332.8	181.7	64.9	101.9	NA	NA	ND	LC-DAD	[71]
	KF 3390	Maize	Poland	29.12	32.40	255.08	138.15	NA	NA	ND	HPLC	[39]
***F. concentricum***	KF 3755	Pineapple	Costa Rica	11.40	8.69	17.33	18.17	NA	NA	312.2	HPLC	[39]
***F. culmorum***	KF 3798	Asparagus	Poland	ND	ND	0.06	ND	NA	NA	ND	HPLC	[39]
***F. equiseti***	KF 3563	Asparagus	Poland	43.47	36.81	29.18	30.39	NA	NA	ND	HPLC	[39]
	KF 3749	Tomato	Poland	39.27	38.18	ND	29.22	NA	NA	ND	HPLC	[39]
	KF 3430	Banana	Ecuador	31.17	32.15	32.98	41.22	NA	NA	ND	HPLC	[39]
	Feq16	Wheat	Italy	ND	≤0.01	≤0.01	≤0.01	NA	NA	≤0.01	LC-DAD	[71]
	Feq136	Wheat	Italy	≤0.01	0.02	≤0.01	0.02	NA	NA	ND	LC-DAD	[71]
***F. fujikuroi***	KF 3631	Rice	Thailand	ND	ND	ND	ND	NA	NA	428.09	HPLC	[39]
***F. globosum***	6646	Maize	South Africa	NA	NA	NA	NA	NA	NA	110	LC-MS	[84]
***F. lactis***	KF 3641	Pepper	Poland	30.97	26.94	ND	ND	NA	NA	ND	HPLC	[39]
***F. nygamai***	KF 337	Pigeon Pea	India	10.45	ND	9.50	ND	NA	NA	22.86	HPLC	[39]
***F. oxysporum***	KF 3567	Garlic	Poland	ND	6.42	8.25	7.28	NA	NA	80.03	HPLC	[39]
	KF 3805	Asparagus	Poland	ND	ND	ND	ND	NA	NA	0.53	HPLC	[39]
***F. poae***	Fp26	Wheat	Italy	≤0.01	0.07	0.03	0.05	NA	NA	3.5	LC-DAD	[71]
	156	Wheat	Italy	≤0.01	0.03	0.03	ND	ND	ND	10.5	LC-MS/MS	[105]
	Fp49	Wheat	Italy	≤0.01	0.1	0.05	0.04	NA	NA	9.4	LC-DAD	[71]
	KF 2576	Maize	Poland	34.31	26.89	28.71	ND	NA	NA	37.53	HPLC	[39]
***F. proliferatum***	KF 3382	Pineapple	Hawaii	ND	ND	ND	ND	NA	NA	3.39	HPLC	[39]
	FPG61_CM	Garlic	Spain	NA	NA	NA	NA	NA	NA	671.80	HPLC	[6]
	KF 3363	Garlic	Poland	ND	ND	ND	ND	NA	NA	45.13	HPLC	[39]
	KF 3792	Asparagus	Poland	ND	0.39	0.13	0.06	NA	NA	0.41	HPLC	[39]
	KF 3584	Rice	Thailand	ND	6.39	12.92	19.64	NA	NA	291.87	HPLC	[39]
	KF 3560	Rhubarb	Poland	ND	ND	ND	ND	NA	NA	149.67	HPLC	[39]
	KF 496	Maize	Italy	ND	5.48	9.61	12.89	NA	NA	ND	HPLC	[39]
***F. sambucinum***	179	Wheat	Italy	ND	ND	ND	ND	ND	ND	10.1	LC-MS/MS	[105]
***F. subglutinans***	1084	Maize	South Africa	NA	NA	NA	NA	NA	NA	700	LC-MS	[84]
***F. sporotrichioides***	KF 3815	Asparagus	Poland	ND	0.09	ND	ND	NA	NA	0.21	HPLC	[39]
	KF 3728	Pea	Poland	12.67	ND	5.99	18.15	NA	NA	5.13	HPLC	[39]
	Fsp50	Wheat	Italy	ND	≤0.01	≤0.01	0.02	NA	NA	13.7	LC-DAD	[71]
	194	Wheat	Italy	ND	ND	ND	ND	ND	ND	6.89	LC-MS/MS	[105]
***F. temperatum***	KF 3321	Pineapple	Costa Rica	27.79	34.39	39.20	29.21	NA	NA	290.97	HPLC	[39]
	RCFT 934	Maize	Argentina	NA	NA	NA	NA	NA	NA	1151	HPLC	[106]
	KF 506	Maize	Poland	ND	ND	15.17	9.88	NA	NA	17.47	HPLC	[39]
***F. tricinctum***	KF 3795	Asparagus	Poland	0.1	0.17	0.28	0.38	NA	NA	0.55	HPLC	[39]
	27B14	Malting barley	Italy	8.45	118	39	124	27	0.13	NA	LC-MS/MS	[104]
	3405	Wheat	Finland	NA	94	690	1200	NA	NA	33	HPLC	[5]
***F. verticillioides***	KF 393	Maize	USA	ND	ND	8.75	12.43	NA	NA	2.34	HPLC	[39]

“ND”—not detected; “NA”—not analyzed.

**Table 4:** Maximum levels [μg/g] of naturally occurring depsipeptides in foods and feeds from different countries.

Sample	Origin	ENN A	ENN A_1_	ENN B	ENN B_1_	ENN B_4_	BEA	Reference
**Asparagus**	Poland	ND	0.05	0.06	ND	NA	0.1	[8]
**Barley**	Italy	ND	ND	ND	≤0.01	0.02	≤0.01	[100]
	Italy	0.02	0.06	0.07	0.07	NA	≤0.01	[104]
	Finland	0.95	2	9.76	5.72	NA	0.02	[1]
	Morocco	ND	220	49	32	NA	5	[107]
	Norway	≤0.01	0.04	0.49	0.17	NA	≤0.01	[108]
	Spain	ND	361.57	21.37	45.94	NA	6.94	[97]
	Tunisia	33.6	149	29.2	31	NA	NA	[96]
**Maize**	Brazil	≤0.01	0.31	≤0.01	≤0.01	NA	0.16	[109]
	Croatia	NA	NA	NA	NA	NA	1.84	[110]
	Denmark	≤0.01	≤0.01	0.58	0.09	NA	0.09	[111]
	Japan	NA	NA	NA	NA	NA	0.03	[112]
	Morocco	ND	445	100	8	NA	59	[107]
	Poland	NA	NA	NA	NA	NA	1.73	[95]
	Serbia	0.02	0.03	≤0.01	0.02	NA	0.14	[7]
	Slovakia	NA	NA	NA	NA	NA	3	[113]
	Spain	ND	813.01	6.31	4.34	NA	9.31	[97]
	Tunisia	ND	29.6	ND	17	NA	NA	[96]
	USA	NA	NA	NA	NA	NA	0.5	[114]
**Oats**	Finland	≤0.01	≤0.01	0.02	≤0.01	NA	0.02	[1]
	Italy	ND	≤0.01	≤0.01	ND	0.05	≤0.01	[100]
	Norway	≤0.01	≤0.01	0.05	0.02	NA	0.02	[108]
**Rice**	Iran	ND	≤0.01	ND	ND	ND	≤0.01	[115]
	Spain	ND	814.42	7.95	ND	NA	11.78	[97]
**Rye**	Finland	ND	≤0.01	0.05	≤0.01	NA	ND	[1]
	Italy	≤0.01	ND	≤0.01	ND	≤0.01	≤0.01	[100]
**Sorghum**	Tunisia	95.6	480	ND	120.1	NA	NA	[96]
**Spelt wheat**	Italy	≤0.01	ND	ND	ND	ND	ND	[100]
**Wheat**	Finland	0.49	0.94	18.3	5.1	NA	≤0.01	[1]
	Italy	≤0.01	≤0.01	0.02	≤0.01	0.04	≤0.01	[100]
	Morocco	0.08	0.13	2.57	0.35	NA	0.02	[116]
	Morocco	34	209	11	19	NA	4	[107]
	Norway	≤0.01	0.02	0.79	0.18	NA	≤0.01	[108]
	Poland	0.27	3.6	28.52	11.8	NA	0.02	[57]
	Romania	0.14	0.36	0.41	0.51	NA	NA	[117]
	Spain	ND	634.85	ND	ND	NA	3.5	[97]
	Tunisia	75.1	177.7	180.6	58.5	NA	NA	[96]
	UK	0.04	0.17	0.13	0.30	NA	NA	[85]
**Breakfast cereals**	Morocco	29.7	688	81.1	795	NA	5.3	[99]
	Spain	ND	268.54	ND	ND	NA	3.12	[97]
	Tunisia	121.3	480	295	120.1	NA	NA	[96]
**Infant cereals**	Morocco	ND	52	5.7	14.5	NA	10.6	[99]
**Pasta**	Italy	≤0.01	≤0.01	0.11	≤0.01	≤0.01	ND	[100]
**Oat flour**	Spain	ND	388.38	ND	ND	NA	4.18	[97]
**Wheat flour**	Japan	≤0.01	0.03	0.63	0.09	NA	≤0.01	[112]
**Corn grits**	Japan	ND	ND	ND	ND	NA	0.03	[112]
**Bovine feed**	Spain	ND	≤0.01	0.04	0.02	NA	0.05	[98]
**Ovine feed**	Spain	ND	≤0.01	0.09	0.03	NA	0.13	[98]
**Caprine feed**	Spain	ND	≤0.01	0.02	≤0.01	NA	0.02	[98]
**Horses feed**	Spain	ND	≤0.01	0.04	≤0.01	NA	0.03	[98]
**Porcine feed**	Finland	0.31	0.55	1.51	1.85	NA	0.41	[102]
	Spain	ND	≤0.01	0.06	0.02	NA	≤0.01	[98]
**Poultry feed**	Brazil	ND	≤0.01	≤0.01	≤0.01	NA	0.02	[109]
	Spain	ND	≤0.01	0.05	0.02	NA	0.02	[98]
	UK	0.04	0.03	2.19	0.40	NA	0.48	[101]
**Rabbits feed**	Spain	ND	≤0.01	0.05	0.02	NA	≤0.01	[98]
**Dogs feed**	Spain	ND	≤0.01	0.02	≤0.01	NA	0.04	[98]
**Cats feed**	Spain	ND	ND	≤0.01	≤0.01	NA	ND	[98]
**Fish feed**	Scotland/Norway/ Spain	≤0.01	≤0.01	0.03	≤0.01	NA	0.08	[103]

“ND”—not detected; “NA”—not analyzed.
